# Impact of *CD40* gene polymorphisms on the risk of cervical squamous cell carcinoma: a case-control study

**DOI:** 10.1186/s12885-023-11367-3

**Published:** 2023-09-11

**Authors:** Manning Zhu, Xiaoying Li, Yanan Feng, Tianshuang Jia, Songxue Li, Liping Gong, Shuang Dong, Xianchao Kong, Litao Sun

**Affiliations:** 1 Cancer Center, Department of Ultrasound Medicine, Zhejiang Provincial People’s Hospital (Affiliated People’s Hospital), Hangzhou Medical College, Hangzhou, Zhejiang China; 2 Department of Ultrasound, The Second Affiliated Hospital of Harbin Medical University, Harbin, Heilongjiang Province China; 3 Department of Obstetrics and Gynecology, The Second Affiliated Hospital of Harbin Medical University, Harbin, Heilongjiang Province China

**Keywords:** Cervical squamous cell carcinoma, CD40, SNPs, Association studies

## Abstract

**Background:**

Cervical cancer is the fourth most common cancer among women worldwide. Genome-wide association studies have revealed multiple susceptible genes and their polymorphisms for cervical cancer risk. Therefore, we aimed to investigate the correlation between single nucleotide polymorphisms (SNPs) of the *CD40* gene and susceptibility to cervical squamous cell carcinoma (CSCC) in a population from the northeastern Han Chinese population.

**Methods:**

The three SNPs (rs1800686, rs3765459, and rs4810485) of the *CD40* gene were analyzed by multiplex polymerase chain reaction (PCR) combined with next-generation sequencing methods in 421 patients with CSCC, 594 patients with high-grade squamous intraepithelial lesions (HSIL), and 504 healthy females. Multivariate logistic regression analysis was used to analyze the potential relationship between *CD40* gene polymorphisms and CSCC, or HSIL.

**Results:**

Our research results showed the AA genotype of rs1800686 had a protective effect on CSCC in comparison to the GG genotype and AG+GG genotypes (AA vs. GG: *p* = 0.0389 and AA vs. AG+GG: *p* = 0.0280, respectively). After FDR correction, the results were still statistically significant (*p* = 0.0389 and *p* = 0.0389, respectively). Similarly, rs3765459 showed a reduced risk association for CSCC in the codominant and recessive models (AA vs. GG: *p* = 0.0286 and AA vs. AG+GG: *p* = 0.0222, respectively). Significant differences remained after FDR correction (*p* = 0.0286 and *p* = 0.0286, respectively). However, these differences were no longer significant after the Bonferroni correction. In addition, the genotypes for the rs4810485 polymorphisms were associated with parity of the patients with CSCC. The genotypes for the rs3765459 polymorphisms were significantly correlated with the D-dimer of the patients with CSCC. The 3 SNPs genotypes of the *CD40* gene were closely related to the squamous cell carcinoma antigen (SCC) of the patients with HSIL.

**Conclusions:**

The *CD40* gene may play a role in the occurrence and development of CSCC.

**Supplementary Information:**

The online version contains supplementary material available at 10.1186/s12885-023-11367-3.

## Introduction

Cervical cancer is one of the leading causes of cancer-related deaths in women. In 2020, it is estimated that 604,127 new cervical cancer cases and 341,831 deaths occurred worldwide [1, 2]. However, in China, there are about 109,741 new cases of cervical cancer and 59,060 deaths. China is still one of the countries with the highest incidence and mortality of cervical cancer [3]. There are studies to indicate that risk factors including various sexual partners, early pregnancy, multiple pregnancies within a limited period of time, oral contraceptive pills, smoking habits, and limited access to health care play a role in cervical cancer development [4, 5]. Persistent human papillomavirus (HPV) infection is the most important risk factor for cervical cancer and squamous intraepithelial lesions (SIL) [6, 7]. However, not all patients infected with HPV will develop cervical cancer, and nearly 70–90% of patients can recover from this infection [8]. More importantly, studies revealed that genetic heritability is one of the most common intrinsic factors that increase the probability of developing cervical cancer by almost 27% [9]. According to an Iranian study, cervical cancer susceptibility is closely related to the interaction of HPV infection and host genetic factors. [10].

HPVs are small, non-enveloped, double-stranded DNA viruses and the most common viral culprit of reproductive tract infections, which belong to the Papillomaviridae family [11–13]. They are classified into low-risk and high-risk HPVs (lr and hrHPVs) based on their oncogenic capacity [11, 14, 15]. lrHPVs (such as HPV 6, 11, 42, 43, and 44) cause benign epithelial lesions, such as verrucae, warts, and papillomas [14, 16, 17]. while hrHPVs (such as HPV 16, 18, 31, 33, 34, 35, 39, 45, 51, 52, 56, 58, 59, 66, 68, and 70) cause cervical, anal, penile, vulval, vaginal, and oropharyngeal cancer [14, 16, 17]. Infection with hrHPVs, such as the most prevalent HPV 16 and 18 subtypes, results in the constitutive expression of their oncogenes E6 and E7 [18]. E6 and E7 oncogenes can inactivate the functions of the tumor suppressor proteins p53 and retinoblastoma protein and can also inhibit inflammatory responses, in turn suppressing host defense mechanisms against infection and cancer [19–21].

CD40 is composed of 277 amino acids and has a molecular weight of 40–50 kDa, which belongs to the tumor necrosis factor receptor (TNFR) superfamily. It is encoded by a gene located on chromosome 20q11–13 and comprises 8 introns and 9 exons [22–24]. CD40 is expressed in a variety of cell types, including normal B lymphocytes, macrophages, endothelial cells, and dendritic cells [25]. In addition to being expressed in normal lymphocytes, CD40 is also present in malignant hematopoietic cells, including leukemias, lymphomas, and solid cancers [26]. Some studies have pointed out that CD40 expression is related to the increased risk of angiogenesis in some tumors [27].

Hill SC et al. [28] found that the expression of CD40 on HPV-infected lesions and advanced cervical squamous cell carcinoma (CSCC) is significantly higher than that of normal cervical tissues. These findings were further confirmed by Huang Q et al. [29]. In vivo studies have shown that the expression of CD40 is correlated with HPV positivity, vascular endothelial growth factor (VEGF) expression, and microvessel density [27, 30]. In recent years, various studies have shown that *CD40* gene polymorphisms are related to some malignant tumors, such as non-Hodgkin’s lymphoma [31], breast cancer [32], and lung cancer [33, 34]. However, there were few reports on the relationship between the *CD40* gene and cervical cancer [35, 36]. Therefore, this study aimed to explore the possible impact of *CD40* gene polymorphisms on the occurrence of CSCC and high-grade squamous intraepithelial lesions (HSIL) in the northeastern Han Chinese population.

## Materials and methods

### Study subjects

The case groups of this case-control study were continuously recruited from the same center (Department of Obstetrics and Gynecology, the Second Affiliated Hospital of Harbin Medical University, Harbin, Heilongjiang Province, China) from September 2014 to October 2018. The case groups included 421 patients with CSCC and 594 patients with HSIL. 101 patients with CSCC and 362 patients with HSIL agreed to obtain cervical brushing exfoliated cells to do HR-HPV detection. 92 were found to be HPV positive and 9 were HPV negative in the CSCC patients, and 341 were found to be HPV positive and 21 were HPV negative in the HSIL patients. Further stratification by HPV sub-type shows that 51 samples were HPV 16 and/or 18 in the CSCC patients and 132 samples were HPV 16 and/or 18 in the HSIL patients. All patients were confirmed by pathology experts of the Second Affiliated Hospital of Harbin Medical University. All the women with benign cervical benign lesions, cervical benign tumors, other cervical malignant tumors, and cervical lesions who had a history of preoperative radiotherapy and chemotherapy and other cancers were excluded from the study.

The 504 cases in the control group were all those who went to the physical examination center of the hospital for routine health examinations at the same time. The control group had no history of cancer or family history of cancer, and the results of the Thinprep cytologic test (TCT) were normal. The results of the routine blood examination and other biochemical indicators were within the reference value range. Patients with any gynecological diseases, a history of gynecological diseases, gynecological surgery, hypoimmunity and immunological diseases, skin or genital condyloma, and other cancers were excluded.

All of the cases and controls agreed with the ethics of the study and signed informed consent, which was approved by the ethics committee of the Second Affiliated Hospital of Harbin Medical University.

### Selection of SNPs

Based on the characteristics of the East Asian population in the dbSNP database (minor allele frequency [MAF] > 15%) and tracking the relevant literature [35, 36], we selected four SNPs of the *CD40* gene. They were rs1800686 (G > A), rs1883832 (T > C), rs3765459 (G > A), and rs4810485 (T > G). Rs1800686 was located in the 508-bp upstream region, rs1883832 was located in the Kozak consensus sequence of the 5´-UTR, rs3765459 was located in the intron 8 of the *CD40* gene, and rs4810485 was located in the intron 1 of the *CD40* gene.

### Extraction and genotyping of DNA

A peripheral venous blood sample (4–5 ml) was collected from each subject into a 2% EDTA-Na2 anticoagulant tube, which was then stored at -80°C until DNA extraction. Genomic DNA from peripheral blood samples was extracted using the TIANamp Genomic DNA Kit supplied by Tiangen Biotech, China. Genotyping of the selected *CD40* gene SNPs (rs1800686, rs1883832, rs3765459, and rs4810485) was tested using multiplex PCR combined with next generation sequencing methods by the Shanghai Bio Wing Applied Biotechnology Company (http://www.biowing.com.cn) [37]. Primer3 online software (version 0.4.0, http://frodo.wi.mit.edu/) was used to amplify primer sequences. The primers used for amplification are as follows: for rs1800686 forward 5’-CACTCTTAATAAATGCCTGTCTCC-3’ and reverse 5’-AGAAAACGGGAAGGCCAC-3’; for rs1883832 forward 5’-CCGCGATTGGTCTTTGAAG-3’ and reverse 5’-CTTTCCTTCTCATTCCCCACTC-3’; for rs3765459 forward 5’-CACTCTGGAAGCTCTTCGTC-3’ and reverse 5’-GAAAATTGATCTCCTGGGGTTC-3’ and for rs4810485 forward 5’-CTCATTCTGGAGGCTGGGAATC-3’ and reverse 5’-ATTGCTTCAGGTGAAAGTGAAAG-3’. The TIANgel Midi Purification Kit (Tiangen Biotech, China) was utilized for purifying the PCR products after PCR amplification was performed. The purified PCR products were sequenced on the Illumina HiSeq XTen platform using paired-end sequencing (2 × 150 bp) as directed by the manufacturer. The Burrows-Wheeler Aligner (BWA, v0.7.12) was used to align the sequences to the human reference genome, and the Samtools (v0.1.19) was used for SNP calling and genotyping [38]. Some samples (73 cases) were randomly selected for blind DNA replication for quality control in genotyping.

### Statistics

The SPSS software package, version 24.0 (SPSS, Institute Inc.), was used to analyze the clinical characteristics between cases and controls. The genotypic distribution of each SNP in control subjects was tested for departure from Hardy-Weinberg equilibrium (HWE) using an exact test. There were six inheritance models, including the allele model, the codominant model, the dominant model, the recessive model, the overdominant model, and the genotypic model. The 95% confidence interval (CI) and odds ratio (OR) were calculated through multiple logistic regression analysis to evaluate the potential association between *CD40* gene polymorphisms and CSCC or HSIL. Empirical *p* values were calculated by 10,000 permutation tests using the Max (T) permutation procedure implemented in PLINK [39]. We used the false discovery rate (FDR) (*p* < 0.05 was statistically significant) and the Bonferroni correction (*p* < 0.016 (0.05/3) was statistically significant) to control for multiple hypothesis testing. SHEsis software was used to implement the Haplotype analysis between groups [40]. For all statistical analyses, *p* < 0.05 was considered statistically significant.

## Results

### Quality control and SNP genotype

All of the tested SNPs were in agreement with the HWE in the control population of this study (*p* > 0.05) except for rs1883832 (*p* = 0.016), and therefore it was excluded from the analysis (Supplementary Table S1). In addition, quality control was set up for the genotypes of several samples. The genotype calling rate in 73 quality control samples was 98.50%, which fully improved the reliability of the follow-up research results.

### Clinical characteristics of the study population

The clinical characteristics of the CSCC group, the HSIL group, and the control group were shown in Table 1. The ages of the patients in the CSCC group and the HSIL group were significantly higher than those in the control group. The ages, menarche ages, fibrinogen (FIB), D-dimer, and squamous cell carcinoma antigen (SCC) of the patients with CSCC were significantly higher than those with HSIL. While the prothrombin time (PT), activated partial thromboplastin time (APTT), and thrombin time (TT) of the patients with CSCC were significantly lower than those with HSIL. The percentages of smoking and amenorrhea were higher in the CSCC group than those in the HSIL group. In addition, there was a significant difference in parity in the CSCC group compared with the HSIL group.

**Table 1:** Clinical characteristics of all subjects in this study

Variable	Controls (n = 504)	CSCC (n = 421)	HSIL (n = 594)	*p* ^a^	*p* ^b^	*p* ^c^
Age (years)	39.00 (32.00–44.00)	49.00 (43.00–56.00)	41.00 (35.00–47.00)	**< 0.0001**	**< 0.0001**	**< 0.0001**
Menarche age (years)	-	15.00 (14.00–16.00)	14.00 (13.00–16.00)	-	-	**< 0.0001**
Amenorrhea n (%)	-	193 (47.1)	103 (17.5)	-	-	**< 0.0001**
Parity (n)	-	1.00 (1.00–2.00)	1.00 (1.00–1.00)	-	-	**< 0.0001**
Abortion n (%)	-	281 (71.7)	390 (70.9)	-	-	0.7957
Smoking n (%)	-	31 (7.4)	25 (4.2)	-	-	**0.0304**
Drinking n (%)	-	5 (1.2)	3 (0.5)	-	-	0.2879
PT (seconds)	-	10.70 (10.20–11.10)	10.80 (10.40–11.30)	-	-	**0.0041**
APTT (seconds)	-	33.80 (31.60–36.10)	34.55 (32.40–37.10)	-	-	**< 0.0001**
FIB (g/l)	-	2.95 (2.61–3.35)	2.68 (2.42–3.06)	-	-	**< 0.0001**
TT (seconds)	-	14.10 (13.20–14.80)	14.30 (13.50–15.20)	-	-	**0.0018**
D-dimer (ng/ml)	-	76.00 (48.00-119.00)	62.00 (40.00–92.00)	-	-	**< 0.0001**
SCC (ng/ml)	-	1.70 (1.00-4.30)	0.80 (0.60–1.10)	-	-	**< 0.0001**

CSCC, cervical squamous cell carcinoma; HSIL, high-grade squamous intraepithelial lesions; PT, prothrombin time; APTT, activated partial thromboplastin time; TT, thrombin time; FIB, fibrinogen; SCC, squamous cell carcinoma antigen; *p*, *p* value

^a^ Comparison between CSCC and controls

^b^ Comparison between HSIL and controls

^c^ Comparison between CSCC and HSIL.

*p* < 0.05 was considered as statistically significant (bold)

### Genotype and allele association analysis

The genotype and allele frequencies of each SNP in the control group and the CSCC group were summarized in Table 2. We found that compared with the control group, rs1800686 was significantly associated with CSCC in the codominant model and the recessive model (AA vs. GG: *p* = 0.0389 and AA vs. AG + GG: *p* = 0.0280, respectively). After FDR correction, the results were still statistically significant (*p* = 0.0389 and *p* = 0.0389, respectively). There were obvious differences in the genotype frequencies of rs3765459 between the CSCC group and the control group in the codominant model and the recessive model (AA vs. GG: *p* = 0.0286 and AA vs. AG + GG: *p* = 0.0222, respectively). Significant differences remained after FDR correction (*p* = 0.0286 and *p* = 0.0286, respectively). After 10,000 permutations, the results were still statistically significant (*p* < 0.05, all). The consistency of 10,000 permutations further supports the reliability of our results. However, these associations did not survive the Bonferroni correction.

**Table 2:** Distribution of 3 SNPs genotypes and alleles of the *CD40* gene in patients with CSCC and controls

SNP	Controls	CSCC	*p* ^a^	Model		*p* ^b^	OR (95% CI)	*p* ^c^	OR (95% CI)	*p* ^d^
rs1800686										
GG	220 (43.9)	185 (44.2)		Allele	A vs. G	0.2814	0.898 (0.738–1.092)	0.1537	0.850 (0.680–1.063)	0.1593
AG	225 (44.9)	206 (49.2)		Codominant	AA vs. GG	**0.0392**	0.595 (0.363–0.974)	**0.0389**	0.561 (0.324–0.971)	**0.0362**
AA	56 (11.2)	28 (6.68)	0.0515		AG vs. GG	0.5400	1.089 (0.830–1.429)	0.9863	0.997 (0.730–1.362)	0.9831
				Dominant	AA + AG vs. GG	0.9289	0.988 (0.761–1.283)	0.5618	0.916 (0.680–1.233)	0.5596
G	665 (66.4)	576 (68.7)		Recessive	AA vs. AG + GG	**0.0200**	0.570 (0.355–0.915)	**0.0280**	0.552 (0.325–0.938)	**0.0258**
A	337 (33.6)	262 (31.3)	0.2804	Overdominant	AG vs. AA + GG	0.2061	1.182 (0.912–1.532)	0.4808	1.112 (0.828–1.494)	0.4982
				Genotypic	AA vs. AG vs. GG	0.2602	0.890 (0.726–1.090)	0.1479	0.843 (0.669–1.063)	0.1417
rs3765459										
GG	219 (43.5)	182 (43.5)		Allele	A vs. G	0.3256	0.906 (0.745–1.103)	0.1128	0.834 (0.666–1.044)	0.1153
AG	231 (45.9)	210 (50.2)		Codominant	AA vs. GG	**0.0423**	0.590 (0.355–0.982)	**0.0286**	0.529 (0.299–0.935)	**0.0233**
AA	53 (10.5)	26 (6.22)	0.0537		AG vs. GG	0.5168	1.094 (0.834–1.435)	0.7964	0.960 (0.703–1.311)	0.7931
				Dominant	AA + AG vs. GG	0.9459	1.009 (0.777–1.310)	0.4816	0.898 (0.667–1.211)	0.4419
G	669 (66.5)	574 (68.7)		Recessive	AA vs. AG + GG	**0.0200**	0.560 (0.344–0.913)	**0.0222**	0.527 (0.304–0.912)	**0.0216**
A	337 (33.5)	262 (31.3)	0.3247	Overdominant	AG vs. AA + GG	0.1668	1.201 (0.926–1.556)	0.5521	1.094 (0.814–1.469)	0.5659
				Genotypic	AA vs. AG vs. GG	0.3229	0.901 (0.734–1.107)	0.1153	0.828 (0.654–1.047)	0.0897
rs4810485										
GG	224 (44.8)	171 (40.9)		Allele	T vs. G	0.4905	1.116 (0.817–1.524)	0.6344	1.088 (0.769–1.538)	0.6368
TG	222 (44.4)	201 (48.1)		Codominant	TT vs. GG	0.6259	1.116 (0.718–1.734)	0.7367	1.088 (0.666–1.776)	0.7345
TT	54 (10.8)	46 (11.0)	0.4768		TG vs. GG	0.2252	1.186 (0.900-1.563)	0.1548	1.261 (0.916–1.737)	0.1682
				Dominant	TT + TG vs. GG	0.2414	1.170(0.900–1.520)	0.2009	1.215(0.902–1.636)	0.1999
G	670(67.0)	543(65.0)		Recessive	TT vs. TG + GG	0.9176	1.022(0.674–1.550)	0.9068	0.972(0.603–1.567)	0.9078
T	330(33.0)	293(35.0)	0.3561	Overdominant	TG vs. TT + GG	0.2725	1.156(0.892–1.499)	0.1782	1.226(0.911–1.649)	0.1871
				Genotypic	TT vs. TG vs. GG	0.3527	1.098(0.902–1.336)	0.3642	1.109(0.887–1.387)	0.3998

CSCC, cervical squamous cell carcinoma; *p*, *p* value

^a^ Comparison between CSCC and controls

^b^ Calculated using multivariate logistic regression analysis

^c^ Adjusted by age

^d^ Adjusted by age and then calculated using 10,000 permutations for each model to correct the multiple test

*p* < 0.05 was considered as statistically significant (bold)

Further stratification by HPV status showed the *CD40* gene SNPs (rs1800686, rs3765459, and rs4810485) were not associated with the risk of HPV infection status (HPV positive, HPV 16 and/or 18, and HPV negative) in the patients with CSCC when compared to controls. Similarly, there were also no significant correlations between the CD40 gene SNPs and the risk of HPV infection status in the patients with HSIL, as shown in Table 3 (*p* > 0.05, all).

**Table 3:** Association of *CD40* gene SNPs (rs1800686, rs3765459, and rs4810485) with case-control status

		rs1800686				rs3765459				rs4810485			
	Stratum	N_cases_	N_controls_	*p*	OR (95% CI)	N_cases_	N_controls_	*p*	OR (95% CI)	N_cases_	N_controls_	*p*	OR (95% CI)
CSCC										418			
	HPV Positive	91	501	0.1568	1.918(0.779–4.724)	91	503	0.1454	1.962(0.792–4.861)	91	500	0.5104	0.792(0.396–1.585)
	HPV 16 and/or 18	51	501	0.9956	1.003(0.401–2.507)	50	503	0.7005	1.214(0.452–3.258)	51	500	0.7071	0.845(0.350–2.036)
	HPV Negative	9	501	0.9710	-	9	503	0.9714	-	9	500	0.9675	-
HSIL													
	HPV Positive	341	501	0.4553	1.191(0.753–1.884)	339	503	0.6233	1.123(0.707–1.784)	335	500	0.3381	1.258(0.786–2.013)
	HPV 16 and/or 18	132	501	0.2406	1.523(0.754–3.073)	132	503	0.6324	1.174(0.608–2.268)	129	500	0.9411	1.024(0.549–1.909)
	HPV Negative	21	501	0.6676	0.760(0.217–2.664)	20	503	0.5986	0.714(0.203–2.506)	21	500	0.8340	1.173(0.265–5.196)

CSCC, cervical squamous cell carcinoma; HSIL, high-grade squamous intraepithelial lesions; *p*, the *p* value was adjusted by age; OR, Odds Ratio for the recessive model with controls as reference group; CI, confidence interval

*p* < 0.05 was considered as statistically significant (bold)

The genotype and allele frequencies of each SNP in the control group and the HSIL group were summarized in Supplementary Table S2. The results showed that the genotype and allele frequencies of rs1800686, rs3765459, and rs4810485 were not significantly different in the HSIL group compared with the control group (*p* > 0.05, all). After 10,000 permutations, the results were still not statistically significant (*p* > 0.05, all).

The genotype and allele frequencies of each SNP in the HSIL group and the CSCC group were analyzed, as shown in Supplementary Table S3. No differences for alleles and genotypes were identified for the comparison of three SNPs (rs1800686, rs3765459, and rs4810485) between the CSCC group and the HSIL group. (*p* > 0.05, all). After 10,000 permutations, there were still no obvious correlation (*p* > 0.05, all).

The 3 SNPs genotypes of the CD40 gene in patients with CSCC and HSIL by clinical features were summarized in Table 4. We found that in the CSCC group, the genotypes for the rs4810485 polymorphisms were associated with parity (*p* = 0.0378), and the genotypes for the rs3765459 polymorphisms were significantly correlated with D-dimer (*p* = 0.0346). The genotypes for the rs1800686, rs3765459, and rs4810485 polymorphisms were closely related to SCC in the HSIL group (*p* = 0.0280, *p* = 0.0199, and *p* = 0.0042, respectively).

**Table 4:** Analysis of 3 SNPs genotypes of the *CD40* gene in patients with CSCC and HSIL by clinical features

Variables	rs1800686								rs3765459								rs4810485							
	CSCC				HSIL				CSCC				HSIL				CSCC				HSIL			
	AA	AG	GG	*p*	AA	AG	GG	*p*	AA	AG	GG	*p*	AA	AG	GG	*p*	TT	TG	GG	*p*	TT	TG	GG	*p*
Smoking				0.7215				0.4789				0.7487				0.3273				0.8019				0.9547
No	26	192	169		50	262	253		23	196	167		48	267	249		43	184	159		56	276	226	
Yes	1	14	16		3	9	13		2	14	15		3	8	13		3	17	11		2	12	11	
Menarche age (years)				0.5874				0.3405				0.8837				0.9149				0.2404				0.3933
< 15	14	91	88		36	157	152		12	95	85		31	160	151		21	85	87		31	165	146	
≥ 15	12	109	89		17	113	114		12	109	89		20	114	111		22	109	78		28	121	91	
Amenorrhea				0.5277				0.2476				0.2445				0.3228				0.3121				0.1495
No	10	100	82		5	51	47		9	106	78		6	54	43		17	100	74		11	59	33	
Yes	16	103	97		48	218	216		15	101	98		45	219	216		26	98	92		47	227	201	
Parity (n)				0.2761				0.4036				0.3740				0.4381				**0.0378**				0.1270
0–1	17	131	101		42	195	197		14	133	100		41	198	195		21	115	112		44	204	185	
≥ 2	8	64	69		7	57	50		9	66	67		7	57	48		21	72	48		14	60	35	
PT (seconds)				0.6162				0.2961				0.5927				0.2887				0.0962				0.7996
8.8–12.8	26	197	169		51	266	262		23	203	166		49	269	259		41	189	162		57	283	232	
> 12.8	0	2	4		1	3	1		0	2	4		1	3	1		2	1	3			2	3	
APTT (seconds)				0.3003				0.4981				0.3222				0.6120				0.8978				0.1216
26–42	26	196	166		52	261	254		23	202	163		50	263	252		42	186	160		53	279	228	
> 42	0	3	7		0	8	9		0	3	7		0	9	8		1	4	5		4	6	7	
FIB (g/l)				0.4218				0.5394				0.3926				0.5326				0.3157				1.0000
2–5	25	197	171		52	269	262		22	203	168		50	272	259		42	189	162		57	284	235	
> 5	1	2	2		0	0	1		1	2	2		0	0	1		1	1	3		0	1	0	
TT (seconds)				0.7154				0.1923				0.7083				0.1862				1.0000				0.7723
10–18	26	196	172		50	265	261		23	202	169		48	268	258		43	188	163		57	280	232	
> 18	0	3	1		2	4	2			3	1		2	4	2		0	2	2		0	5	3	
D-dimer (ng/ml)				0.3928				0.7503				**0.0346**				0.6788				0.7588				0.3021
≤ 243	22	187	160		51	262	259		18	195	156		49	265	256		41	175	154		57	281	228	
> 243	3	11	12		1	7	4		4	9	13		1	7	4		2	14	9			4	7	
SCC (ng/ml)				0.6335				**0.0280**				0.8547				**0.0199**				0.4786				**0.0042**
≤ 1.5	9	88	72		39	190	175		10	89	70		37	194	172		22	84	63		31	199	170	
> 1.5	17	110	96		1	25	36		15	111	96		1	24	36		23	104	95		12	32	17	
HPV				0.8496				0.3613				0.8481				0.4096				0.7835				0.1730
Negative	0	6	3		3	7	11		0	6	3		3	7	10		0	5	4		2	6	13	
Positive	6	49	36		32	165	144		6	48	37		32	165	142		13	45	33		30	161	144	
Lymph node metastasis				0.3664								0.4705								0.5248				
No	12	108	87						11	110	86						24	102	81					
Yes	6	32	21						5	33	21						4	29	26					

CSCC, cervical squamous cell carcinoma; HSIL, high-grade squamous intraepithelial lesions; PT, prothrombin time; APTT, activated partial thromboplastin time; TT, thrombin time; FIB, fibrinogen; SCC, squamous cell carcinoma antigen; *p*, *p* value

*p* < 0.05 was considered as statistically significant (bold)

Tables 5 and 6 summarized the genotypic associations of three SNPs with risk factors related to CSCC and HSIL, respectively. For rs4810485 in the CSCC group, the parity between patients with the T allele and the G allele was different (*p* = 0.0138), and the parity between patients with the TT + TG genotype and the GG genotype was different (*p* = 0.0338). For rs3765459 in the CSCC group, the D-dimer between patients with the AA genotype and the AG + GG genotype was different (*p* = 0.0326). In the HSIL group, rs4810485 was significantly associated with parity in the dominant model and the genotypic model (*p* = 0.0237 and *p* = 0.0417, respectively). The 3 SNPs genotypes of the *CD40* gene were significantly correlated with SCC in the HSIL group, and the allele, codominant, dominant, and genotypic models confirmed the credibility of that conclusion, respectively (*p* < 0.05, all).

**Table 5:** Genetypic association between 3 SNPs and CSCC-related risk factors

Model	CSCC	Smoking	Menarche	Amenorrhe	Parity	PT	APTT	FIB	TT	D-dimer	SCC	HPV
		*p*	*p*	*p*	*p*	*p*	*p*	*p*	*p*	*p*	*p*	*p*
rs1800686												
Allele	A vs. G	0.3508	0.7978	1.0000	0.1688	0.2779	0.1208	0.5642	0.7157	0.8345	0.7831	0.9875
Codominant	AA vs. GG	0.3920	0.6949	0.4826	0.4140	0.9738	0.9654	0.3226	0.9707	0.3824	0.4294	0.9658
	AG vs. GG	0.4929	0.4133	0.5007	0.1245	0.3319	0.1466	0.8880	0.4038	0.5730	0.7604	0.6034
Dominant	AA + AG vs. GG	0.3796	0.4640	0.6521	0.1162	0.2614	0.1002	0.8827	0.4716	0.7634	0.8511	0.7337
Recessive	AA vs. AG + GG	0.4606	0.5142	0.3657	0.6502	0.9775	0.9710	0.2498	0.9724	0.2647	0.3523	0.9740
Overdominant	AG vs. AA + GG	0.6141	0.2953	0.3734	0.1816	0.4132	0.2081	0.6470	0.3432	0.3965	0.5156	0.4817
Genotypic	AA vs. AG vs. GG	0.3031	0.7359	0.9971	0.1390	0.2341	0.0892	0.5333	0.6954	0.8319	0.8171	0.9716
rs3765459												
Allele	A vs. G	0.7051	0.8363	0.6411	0.3838	0.2779	0.1208	0.5642	0.7157	0.8345	0.8838	0.9748
Codominant	AA vs. GG	0.9674	0.9159	0.5283	0.9279	0.9750	0.9670	0.2820	0.9721	0.1156	0.8377	0.9662
	AG vs. GG	0.5531	0.6580	0.1790	0.1688	0.3052	0.1282	0.8507	0.4274	0.1860	0.6545	0.5589
Dominant	AA + AG vs. GG	0.5558	0.6757	0.3326	0.1673	0.2465	0.0912	0.9094	0.4882	0.4295	0.6740	0.6878
Recessive	AA vs. AG + GG	0.9029	0.8123	0.3358	0.7652	0.9789	0.9728	0.2049	0.9741	**0.0326**	0.7231	0.9740
Overdominant	AG vs. AA + GG	0.5213	0.5988	0.1575	0.1320	0.3744	0.1779	0.6001	0.3705	0.0738	0.5559	0.4455
Genotypic	AA vs. AG vs. GG	0.6577	0.7990	0.6698	0.3002	0.2247	0.0830	0.5245	0.6892	0.8283	0.8387	0.9858
rs4810485												
Allele	T vs. G	0.7363	0.2713	0.9152	**0.0138**	0.6091	0.6572	0.7552	0.5669	0.8552	0.2372	0.3531
Codominant	TT vs. GG	0.9661	0.9608	0.5166	0.0565	0.2314	0.7299	0.5887	0.9699	0.5528	0.1317	0.9624
	TG vs. GG	0.4440	0.1044	0.1600	0.4333	0.1634	0.6265	0.2450	0.9237	0.5191	0.6474	0.7043
Dominant	TT + TG vs. GG	0.5570	0.0905	0.4040	**0.0338**	0.6624	0.5710	0.4026	0.7222	0.4911	0.2480	0.6117
Recessive	TT vs. TG + GG	0.8135	0.8694	0.2969	0.0521	0.0978	0.9382	0.5109	0.9763	0.5965	0.4308	0.9614
Overdominant	TG vs. TT + GG	0.4693	0.0775	0.1443	0.3804	0.1592	0.6097	0.2395	0.9359	0.3146	0.5279	0.7508
Genotypic	TT vs. TG vs. GG	0.7422	0.2297	0.8907	0.0117	0.6026	0.6423	0.7441	0.5522	0.7899	0.2147	0.3312

CSCC, cervical squamous cell carcinoma; PT, prothrombin time; APTT, activated partial thromboplastin time; TT, thrombin time; FIB, fibrinogen; SCC, squamous cell carcinoma antigen; *p*, *p* value

*p* < 0.05 was considered as statistically significant (bold)

**Table 6:** Genetypic association between 3 SNPs and HSIL-related risk factors

Model	HSIL	Smoking	Menarche	Amenorrhe	Parity	PT	APTT	FIB	TT	D-dimer	SCC	HPV
		*p*	*p*	*p*	*p*	*p*	*p*	*p*	*p*	*p*	*p*	*p*
rs1800686												
Allele	A vs. G	0.7627	0.2664	0.4087	0.7737	0.2294	0.2896	0.9554	0.1274	0.5555	**0.0120**	0.7264
Codominant	AA vs. GG	0.8140	0.1474	0.1381	0.3368	0.2499	0.9558	0.9670	0.1024	0.8323	**0.0430**	0.7632
	AG vs. GG	0.3629	0.8138	0.7461	0.5181	0.3495	0.7691	0.9591	0.4361	0.3865	0.1113	0.2684
Dominant	AA + AG vs. GG	0.4693	0.5823	0.8524	0.8003	0.2888	0.4995	0.9569	0.2706	0.4210	**0.0290**	0.4646
Recessive	AA vs. AG + GG	0.5873	0.1421	0.1114	0.2479	0.3972	0.9708	0.9760	0.1293	0.9470	0.0675	0.3641
Overdominant	AG vs. AA + GG	0.3033	0.7654	0.4532	0.3588	0.5408	0.9455	0.9612	0.8304	0.4007	0.3066	0.1861
Genotypic	AA vs. AG vs. GG	0.7483	0.2774	0.3833	0.7471	0.2157	0.2709	0.9507	0.1166	0.5471	**0.0088**	0.7171
rs3765459												
Allele	A vs. G	0.6615	0.7102	0.9670	0.8729	0.2300	0.4876	0.9554	0.1279	0.5572	**0.0090**	0.8683
Codominant	AA vs. GG	0.7850	0.6766	0.3893	0.4056	0.2419	0.9588	0.9674	0.0966	0.8128	**0.0469**	0.6767
	AG vs. GG	0.2253	0.8584	0.3432	0.4773	0.3597	0.8792	0.9588	0.4517	0.4067	0.0637	0.2684
Dominant	AA + AG vs. GG	0.4275	0.7439	0.5930	0.6161	0.2974	0.8208	0.9567	0.2819	0.4410	**0.0219**	0.4646
Recessive	AA vs. AG + GG	0.5401	0.7152	0.2605	0.2754	0.3760	0.9713	0.9765	0.1164	0.9803	0.0766	0.5909
Overdominant	AG vs. AA + GG	0.2587	0.9048	0.2416	0.2631	0.5660	0.6251	0.9608	0.8686	0.4347	0.2299	0.3363
Genotypic	AA vs. AG vs. GG	0.7255	0.6768	0.9316	0.9233	0.2134	0.4598	0.9506	0.1148	0.5497	**0.0074**	0.9408
rs4810485												
Allele	T vs. G	0.6877	0.1934	0.1313	0.0664	0.3416	0.4127	0.6517	0.7784	0.1135	**0.0044**	0.2214
Codominant	TT vs. GG	0.6929	0.2054	0.3555	0.1464	0.9580	0.1631	-	0.9580	0.9574	**0.0014**	0.6998
	TG vs. GG	0.7915	0.3646	0.0536	0.0612	0.5101	0.5274	0.9617	0.6609	0.2247	0.1350	0.4889
Dominant	TT + TG vs. GG	0.6795	0.1930	0.0754	**0.0237**	0.3747	0.9269	0.9642	0.8799	0.2028	**0.0326**	0.9094
Recessive	TT vs. TG + GG	0.7581	0.3391	0.7649	0.4987	0.9748	0.0628	0.9748	0.9681	0.9742	**0.0041**	0.0841
Overdominant	TG vs. TT + GG	0.8247	0.4825	0.1173	0.0706	0.6502	0.2201	0.9595	0.4871	0.5536	0.6885	0.0771
Genotypic	TT vs. TG vs. GG	0.6446	0.1490	0.1320	**0.0417**	0.3078	0.4047	0.6380	0.7504	0.1324	**0.0027**	0.1880

HSIL, high-grade squamous intraepithelial lesions; PT, prothrombin time; APTT, activated partial thromboplastin time; TT, thrombin time; FIB, fibrinogen; SCC, squamous cell carcinoma antigen; *p*, *p* value

*p* < 0.05 was considered as statistically significant (bold)

### Haplotype analysis

Considering the linkage disequilibrium of genes, haplotype analysis for *CD40* gene polymorphisms was performed (Supplementary Table S4). Results showed that there were no significant differences between the CSCC group and the control group, the HSIL group and the control group, and the CSCC group and the HSIL group, respectively (*p* > 0.05, all).

## Discussion

The incidence rate of cervical cancer has been kept high in China. The morbidity age has shown a younger trend in recent years. Cervical cancer susceptibility is closely linked to host genetic factors. As a host genetic factor, genetic variation at susceptibility loci may have a significant impact on the risk of cervical cancer [41]. Previous studies on *CD40* gene polymorphisms and cervical cancer have been very limited. The association of *CD40* gene polymorphisms with the risk of CSCC development in the northeastern Han Chinese population was first investigated in our study. With further study of the *CD40* gene, it may be a useful biomarker for evaluating the risk of developing cervical cancer and may also be used as a target for therapy.

These two roles of CD40—promoting immune responses and angiogenesis—have opposing effects on the development of a tumor. Immune responses that are tailored to the tumor prevent tumor growth, whereas enhanced angiogenesis promotes tumor growth by giving the tumor cells nutrition and a path to distant organs [42]. Cervical cancer is more dependent on angiogenesis than other cancers [43]. Angiogenesis, the development of new blood vessels, is essential for the initiation of tumors, their growth, and metastasis [44]. The process of tumor-related angiogenesis is regulated by various pro-angiogenic factors, such as VEGF and interleukin 6 (IL-6), which are the dominant regulators of the proliferation of endothelial cells and the formation of new blood vessels [45, 46]. The activation of CD40 can upregulate VEGF, IL-6, and other factors through MAPK, PI3K/Akt, and other signal transduction pathways and then promote tumor angiogenesis by inhibiting endothelial cell apoptosis and promoting the growth of vascular endothelial cells, so as to promote the occurrence and development of cervical cancer [44, 47, 48]. Zhang et al. [49] reported that some SNPs in the coding region can change the amino acid sequence of proteins, while some SNPs in the non-coding region may still affect gene splicing, transcription factor binding, messenger RNA degradation, or the sequence of non-coding RNA. *CD40* gene SNPs can affect the transcription and translation efficiency of CD40, which can determine the effective expression of CD40.

In this study, we investigated the potential effects of rs1800686, rs3765459, and rs4810485 polymorphisms on the occurrence of CSCC and HSIL. Our results showed that rs1800686 and rs3765459 were associated with the risk of CSCC. To date, no study has discussed the association of the rs1800686, rs3765459, and rs4810485 polymorphisms with CSCC risk. Rs1800686 and rs3765459 may be closely related to the pathological classification of cervical cancer. The AA genotype of rs1800686 had a protective effect on CSCC in comparison to the AG + GG genotypes in the recessive model, and these data were statistically significant (CSCC vs. control: OR = 0.552, 95% CI = 0.325–0.938, *p* = 0.0280). Similarly, rs3765459 showed a reduced risk association for CSCC in the recessive model (CSCC vs. control: OR = 0.527, 95% CI = 0.304–0.912, *p* = 0.022). The same results appeared in the codominant model (AA vs. GG). Even after 10,000 permutations and FDR correction, the associations held (*p* < 0.05). Although the interaction effect was not significant after Bonferroni correction, it also suggested a high potential interaction effect as Bonferroni correction was a conservative and stringent correction test. Studies in larger patient cohorts would contribute to elucidating this interaction. Rs1800686 is located in the 5’ near-gene region of the *CD40* gene, where mutations can modulate CD40 promoter activity. Rs3765459 is located in intron 8 of the *CD40* gene, where mutations can lead to abnormal splicing. Therefore, the AA genotypes of rs1800686 and rs3765459 may inhibit CD40 expression. The decrease in CD40 expression levels can down-regulate VEGF, IL-6, and other factors, reducing the risk of CSCC by promoting endothelial cell apoptosis, inhibiting the growth of vascular endothelial cells, and inhibiting tumor angiogenesis. Shuang et al. [32] demonstrated that the rs1800686 and rs3765459 AA genotypes may increase the risk of breast cancer. They observed that the rs1800686 and rs3765459 AA genotypes were higher in patients with breast cancer compared with the control group. A possible explanation for the inconsistencies between the rs1800686 and rs3765459 polymorphisms and different types of cancer is that these polymorphisms might have different genetic effects on different diseases. Similar results can be seen in the studies of Chen et al. [50] and Yi et al. [51], in which they found that the impacts of genetic polymorphisms may be different according to the types of cancer.

Yan et al. [36] reported the association between *CD40* gene polymorphisms and cervical cancer in the Yunnan Han population, pointing out that there was no significant association between rs4810485 and cervical cancer. Purushotham Krishnappa et al. [35] pointed out that rs1800686 and rs4810485 were not related to the occurrence of cervical cancer in the Malaysian population; however, for rs3765459, the G allele increased the risk of cervical cancer (*p* = 0.010). In our study, we discovered no significant associations between the three SNPs of the CD40 gene and the risk of HSIL. The same results appeared in the comparison of CSCC and HSIL women. The reasons for these negative results remain unknown, but two possibilities should be considered. First, it may be because of genetic trait differences; as we know, genetic polymorphisms in human genes are distinct in different ethnicities, populations, and geographic regions. In addition, even though we might find a potential link between the disease-causing gene and the disease itself, cervical cancer is a multi-factorial disease, and individual exposure to diverse environmental factors and genetic backgrounds may cause different results.

Rs4810485 had no significant correlation with the genetic susceptibility of CSCC (*p* > 0.05). However, in the CSCC group, the genotypes for the rs4810485 polymorphisms were associated with parity. The genotypes for the rs3765459 polymorphisms were significantly correlated with the D-dimer of the patients with CSCC. The 3 SNPs genotypes of the *CD40* gene were significantly correlated with the SCC of the patients with HSIL, suggesting that the *CD40* gene polymorphisms genotypes may affect the level of SCC in the patients with HSIL, and further researches are needed.

Studies conducted in vivo and in vitro found that, in contrast to healthy cervical epithelium and non-cancerous keratinocyte cell lines transfected with HPV-DNA, oncogenic HPV positive cervical cancer and HPV-positive cervical cancer cell lines expressed CD40 at high levels [28, 52]. This disparity may explain why HPV infection persists by evading the immune system. The reason cervical cancer has a bad prognosis when the HPV-18 genotype is present may be due to this immune evasion [53]. Unfortunately, we did not find an interaction between the *CD40* gene SNPs (rs1800686, rs3765459, and rs4810485) and the risk of HPV infection status (HPV positive, HPV 16 and/or 18, and HPV negative) in CSCC patients; more samples are required in future studies.

Since *CD40* gene polymorphisms may correlate with CSCC susceptibility, it is convinced that clarification of how polymorphisms of the *CD40* gene have affected mRNA expression and protein expression and how the protein performs in the pathogenesis of the disease is meaningful and helpful. It is widely accepted that mRNA may be regulated by SNPs [54]. Therefore, the mutation of the *CD40* gene may affect the expression of CD40 and play an anti-tumor role. In our study, we did not discuss the association between these polymorphisms and CD40 expression in CSCC and HSIL. Functional studies in the future are necessary to elucidate how polymorphisms regulate CD40 expression, especially their role in cervical cancer pathogenesis.

There were some limitations in the present study. First of all, we found the *CD40* gene SNPs (rs1800686, rs3765459, and rs4810485) with specific HPV genotypes did not influence the patients’ susceptibility towards the developments of HSIL or CSCC; however, the conclusion is to proceed with caution because only 463 patients (45.6%) underwent HR-HPV detection. Second, the association between *CD40* gene polymorphism and its expression level was not investigated, which many previous reports have confirmed. Third, cervical cancer is a complex disease affected by multiple genes, but we only studied the limited sites of the *CD40* gene, and other functional genes and SNPs have not been explored. Therefore, the role of *CD40* and other genes in the occurrence and development of cervical cancer remains to be further studied.

## Conclusion

In conclusion, the study presented is the first to analyze the *CD40* gene polymorphisms in a northeastern Han Chinese population with CSCC and HSIL, which revealed that rs1800686 and rs3765459 were significantly associated with the decreased risk of CSCC. In addition, the genotypes for the rs4810485 polymorphisms were associated with parity of the patients with CSCC. The genotypes for the rs3765459 polymorphisms were significantly correlated with the D-dimer of the patients with CSCC. The 3 SNPs genotypes of the *CD40* gene were closely related to the SCC of the patients with HSIL. Despite the fact that interactions became insignificant after Bonferroni correction, these findings provide novel evidence for risk assessment and personalized intervention in Chinese women with CSCC.

### Electronic supplementary material

Below is the link to the electronic supplementary material.

Supplementary Material 1

## Data Availability

The datasets generated and analyzed during the current study are available from the corresponding author on reasonable request.

## Data Availability

SNP date is available in the Figshare database (https://figshare.com/), 10.6084/m9.figshare.19687518.v1.
