# Supplementary material for: Impact of CD40 gene polymorphisms on the risk of cervical squamous cell carcinoma: a case-control study
Source: BMC Cancer. 2023 Sep 11;23:845. doi: 10.1186/s12885-023-11367-3 (PMC10494347; doi:10.1186/s12885-023-11367-3)
Supplement: Supplementary file 1 — Supplementary Material 1 [file 12885_2023_11367_MOESM1_ESM.doc]

Supplementary Table S1 Hardy–Weinberg Equilibrium test results of *CD40* gene SNPs in the control population

| **Single nucleotide polymorphisms** | **Controls** | |
| --- | --- | --- |
|  | **χ^2^** | ***p*** |
| rs1800686 | 0.018 | 0.893 |
| rs1883832 | 5.853 | 0.016 |
| rs3765459 | 0.476 | 0.490 |
| rs4810485 | 0.008 | 0.927 |

Supplementary Table S2 Distribution of 3 SNPs genotypes and alleles of the *CD40* gene in patients with HSIL and controls

| **SNP** | **Controls** | **HSIL** | ***p*^a^** | **Model** |  | ***p*^b^** | **OR (95% CI)^b^** | ***p*^c^** | **OR (95% CI)^c^** | ***p*^d^** |
| --- | --- | --- | --- | --- | --- | --- | --- | --- | --- | --- |
| rs1800686 |  |  |  |  |  |  |  |  |  |  |
| GG | 220 (43.9) | 267 (45.1) |  | Allele | A vs. G | 0.4202 | 0.929 (0.777-1.111) | 0.4544 | 0.934 (0.780-1.118) | 0.4363 |
| AG | 225 (44.9) | 271 (45.8) |  | Codominant | AA vs. GG | 0.2765 | 0.795 (0.525-1.202) | 0.3264 | 0.812 (0.536-1.231) | 0.2869 |
| AA | 56 (11.2) | 54 (9.12) | 0.5297 |  | AG vs. GG | 0.9527 | 0.992 (0.772-1.276) | 0.8853 | 0.982 (0.762-1.264) | 0.8894 |
|  |  |  |  | Dominant | AA+AG vs. GG | 0.6661 | 0.949 (0.747-1.205) | 0.6561 | 0.947 (0.745-1.204) | 0.6671 |
| G | 665 (66.4) | 805 (68.0) |  | Recessive | AA vs. AG+GG | 0.2673 | 0.800 (0.539-1.187) | 0.3426 | 0.825 (0.555-1.227) | 0.2993 |
| A | 337 (33.6) | 379 (32.0) | 0.4206 | Overdominant | AG vs. AA+GG | 0.8110 | 1.030 (0.811-1.307) | 0.8975 | 1.016 (0.799-1.291) | 0.8222 |
|  |  |  |  | Genotypic | AA vs. AG vs. GG | 0.4009 | 0.925 (0.771-1.109) | 0.4379 | 0.930 (0.775-1.117) | 0.4167 |
| rs3765459 |  |  |  |  |  |  |  |  |  |  |
| GG | 219 (43.5) | 263 (44.6) |  | Allele | A vs. G | 0.4928 | 0.939 (0.785-1.123) | 0.4780 | 0.937 (0.782-1.122) | 0.4931 |
| AG | 231 (45.9) | 275 (46.6) |  | Codominant | AA vs. GG | 0.3484 | 0.817 (0.535-1.247) | 0.3773 | 0.826 (0.540-1.263) | 0.3911 |
| AA | 53 (10.5) | 52 (8.81) | 0.6271 |  | AG vs. GG | 0.9455 | 0.991 (0.772-1.273) | 0.8243 | 0.972 (0.755-1.251) | 0.8143 |
|  |  |  |  | Dominant | AA+AG vs. GG | 0.7841 | 0.967 (0.761-1.228) | 0.7073 | 0.955 (0.751-1.215) | 0.6846 |
| G | 669 (66.5) | 801 (67.9) |  | Recessive | AA vs. AG+GG | 0.3232 | 0.816 (0.546-1.221) | 0.3799 | 0.834 (0.556-1.250) | 0.3791 |
| A | 337 (33.5) | 379 (32.1) | 0.4931 | Overdominant | AG vs. AA+GG | 0.7562 | 1.038 (0.818-1.317) | 0.8846 | 1.018 (0.801-1.293) | 0.9271 |
|  |  |  |  | Genotypic | AA vs. AG vs. GG | 0.5083 | 0.940 (0.782-1.129) | 0.4910 | 0.937 (0.779-1.127) | 0.4923 |
| rs4810485 |  |  |  |  |  |  |  |  |  |  |
| GG | 224 (44.8) | 238 (40.7) |  | Allele | T vs. G | 0.8509 | 1.028 (0.769-1.376) | 0.8929 | 1.020 (0.762-1.366) | 0.5271 |
| TG | 222 (44.4) | 288 (49.2) |  | Codominant | TT vs. GG | 0.8943 | 1.028 (0.681-1.552) | 0.9241 | 1.020 (0.675-1.541) | 0.7139 |
| TT | 54 (10.8) | 59 (10.1) | 0.2804 |  | TG vs. GG | 0.1217 | 1.221 (0.948-1.572) | 0.1280 | 1.219 (0.945-1.572) | 0.1233 |
|  |  |  |  | Dominant | TT+TG vs. GG | 0.1433 | 1.197 (0.941-1.522) | 0.1572 | 1.191 (0.935-1.516) | 0.1481 |
| G | 670 (67.0) | 764 (65.3) |  | Recessive | TT vs. TG+GG | 0.6704 | 0.919 (0.622-1.357) | 0.5807 | 0.895 (0.604-1.326) | 0.7624 |
| T | 330 (33.0) | 406 (34.7) | 0.4042 | Overdominant | TG vs. TT+GG | 0.0882 | 1.230 (0.969-1.561) | 0.0830 | 1.236 (0.973-1.570) | 0.1058 |
|  |  |  |  | Genotypic | TT vs. TG vs. GG | 0.3620 | 1.089 (0.907-1.307) | 0.4136 | 1.080 (0.898-1.298) | 0.3633 |

HSIL, high-grade squamous intraepithelial lesions; *p*, *p* value.

^a^ Comparison between HSIL and controls.

^b^ Calculated using multivariate logistic regression analysis

^c^ Adjusted by age.

^d^ Adjusted by age and then calculated using 10,000 permutations for each model to correct the multiple test.

*p* < 0.05 was considered as statistically significant (bold)

Supplementary Table S3 Distribution of 3 SNPs genotypes and alleles of the *CD40* gene in patients with CSCC and HSIL

| **SNP** | **HSIL** | **CSCC** | ***p*^a^** | **Model** |  | ***p*^b^** | **OR (95% CI)^b^** | ***p*^c^** | **OR (95% CI)^c^** | ***p*^d^** |
| --- | --- | --- | --- | --- | --- | --- | --- | --- | --- | --- |
| rs1800686 |  |  |  |  |  |  |  |  |  |  |
| GG | 267 (45.1) | 185 (44.2) |  | Allele | A vs. G | 0.7231 | 0.966 (0.799-1.169) | 0.6499 | 1.060 (0.823-1.366) | 0.6172 |
| AG | 271 (45.8) | 206 (49.2) |  | Codominant | AA vs. GG | 0.2496 | 0.748 (0.457-1.226) | 0.9047 | 0.960 (0.494-1.867) | 0.9237 |
| AA | 54 (9.12) | 28 (6.68) | 0.2944 |  | AG vs. GG | 0.4861 | 1.097 (0.845-1.424) | 0.4189 | 1.155 (0.815-1.637) | 0.4645 |
|  |  |  |  | Dominant | AA+AG vs. GG | 0.7507 | 1.042 (0.810-1.339) | 0.4602 | 1.136 (0.810-1.595) | 0.5012 |
| G | 805 (68.0) | 576 (68.7) |  | Recessive | AA vs. AG+GG | 0.1615 | 0.712 (0.443-1.145) | 0.8306 | 0.933 (0.492-1.767) | 0.7816 |
| A | 379 (32.0) | 262 (31.3) | 0.7228 | Overdominant | AG vs. AA+GG | 0.2787 | 1.148 (0.894-1.474) | 0.3967 | 1.156 (0.826-1.619) | 0.3671 |
|  |  |  |  | Genotypic | AA vs. AG vs. GG | 0.7200 | 0.964 (0.790-1.177) | 0.6168 | 1.072 (0.817-1.405) | 0.6392 |
| rs3765459 |  |  |  |  |  |  |  |  |  |  |
| GG | 263 (44.6) | 182 (43.5) |  | Allele | A vs. G | 0.7117 | 0.965 (0.797-1.167) | 0.8278 | 1.029 (0.798-1.326) | 0.8166 |
| AG | 275 (46.6) | 210 (50.2) |  | Codominant | AA vs. GG | 0.2092 | 0.723 (0.435-1.200) | 0.4790 | 0.777 (0.387-1.561) | 0.5293 |
| AA | 52 (8.81) | 26 (6.22) | 0.2398 |  | AG vs. GG | 0.4591 | 1.103 (0.850-1.432) | 0.4758 | 1.136 (0.800-1.615) | 0.4684 |
|  |  |  |  | Dominant | AA+AG vs. GG | 0.7409 | 1.043 (0.811-1.342) | 0.5731 | 1.103 (0.785-1.550) | 0.5871 |
| G | 801 (67.9) | 574 (68.7) |  | Recessive | AA vs. AG+GG | 0.1305 | 0.686 (0.421-1.118) | 0.5703 | 0.826 (0.427-1.597) | 0.4799 |
| A | 379 (32.1) | 262 (31.3) | 0.7114 | Overdominant | AG vs. AA+GG | 0.2551 | 1.156 (0.900-1.484) | 0.3918 | 1.159 (0.827-1.624) | 0.2781 |
|  |  |  |  | Genotypic | AA vs. AG vs. GG | 0.6983 | 0.961 (0.785-1.176) | 0.8315 | 1.030 (0.784-1.354) | 0.8089 |
| rs4810485 |  |  |  |  |  |  |  |  |  |  |
| GG | 238 (40.7) | 171 (40.9) |  | Allele | T vs. G | 0.5994 | 1.085 (0.799-1.474) | 0.6470 | 1.101 (0.729-1.665) | 0.8269 |
| TG | 288 (49.2) | 201 (48.1) |  | Codominant | TT vs. GG | 0.7103 | 1.085 (0.704-1.673) | 0.7461 | 1.101 (0.614-1.975) | 0.8332 |
| TT | 59 (10.1) | 46 (11.0) | 0.8757 |  | TG vs. GG | 0.8308 | 0.971 (0.744-1.268) | 0.6045 | 0.910 (0.638-1.299) | 0.4607 |
|  |  |  |  | Dominant | TT+TG vs. GG | 0.8602 | 0.977 (0.758-1.261) | 0.6570 | 0.926 (0.659-1.301) | 0.5796 |
| G | 764 (65.3) | 543 (65.0) |  | Recessive | TT vs. TG+GG | 0.6087 | 1.112 (0.740-1.672) | 0.6109 | 1.155 (0.663-2.012) | 0.6678 |
| T | 406 (34.7) | 293 (35.0) | 0.8722 | Overdominant | TG vs. TT+GG | 0.6279 | 0.940 (0.732-1.207) | 0.4590 | 0.881 (0.630-1.232) | 0.4421 |
|  |  |  |  | Genotypic | TT vs. TG vs. GG | 0.9141 | 1.011 (0.833-1.226) | 0.9179 | 0.986 (0.760-1.280) | 0.8931 |

CSCC, cervical squamous cell carcinoma; HSIL, high-grade squamous intraepithelial lesions; *p*, *p* value.

^a^ Comparison between CSCC and HSIL.

^b^ Calculated using multivariate logistic regression analysis

^c^ Adjusted by age, menarche age, amenorrhea, parity, smoking, PT, APTT, FIB, TT, D dimer, and SCC.

^d^ Adjusted byage, menarche age, amenorrhea, parity, smoking, PT, APTT, FIB, TT, D dimer, and SCC, then calculated using 10,000 permutations for each model to correct the multiple test.

*p* < 0.05 was considered as statistically significant (bold)

Supplementary Table S4 The distribution of the haplotypes constructed by SNPs in *CD40* gene

| **Haplotype** | **Freq (controls)** | **Freq (CSCC)** | **Freq (HSIL)** | ***P*^a^** | **OR^a^ (95% CI)** | ***P*^b^** | **ORb (95% CI)** | ***P*^c^** | **OR^c^ (95% CI)** |
| --- | --- | --- | --- | --- | --- | --- | --- | --- | --- |
| A A G | 0.327 | 0.302 | 0.306 | 0.248 | 0.889(0.728~1.085) | 0.360 | 0.918 (0.764~1.102) | 0.746 | 0.968 (0.797~1.176) |
| G G G | 0.328 | 0.337 | 0.330 | 0.696 | 1.040(0.854~1.266) | 0.800 | 1.024 (0.854~1.227) | 0.870 | 1.016 (0.840~1.228) |
| G G T | 0.324 | 0.341 | 0.334 | 0.453 | 1.078 (0.886~1.312) | 0.514 | 1.062(0.886~1.274) | 0.878 | 1.015 (0.840~1.227) |

CSCC, cervical squamous cell carcinoma; HSIL, high-grade squamous intraepithelial lesions; *p*, *p* value.

^a^ Comparison between CSCC and controls.

^b^ Comparison between HSIL and controls.

^c^ Comparison between CSCC and HSIL.

*p* < 0.05 was considered as statistically significant (bold).
